# Selective Detection of Formaldehyde and Nitrogen Dioxide Using Innovative Modeling of SnO_2_ Surface Response to Pulsed Temperature Profile

**DOI:** 10.3390/s24247964

**Published:** 2024-12-13

**Authors:** Emilie Bialic, Jimmy Leblet, Aymen Sendi, Paul Gersberg, Axel Maupoux, Nicolas Lassabe, Philippe Menini

**Affiliations:** 1 Capgemini Engineering Research and Development, 31000 Toulouse, France; paul.gersberg@capgemini.com (P.G.); axel.maupoux@capgemini.com (A.M.); nicolas.lassabe@capgemini.com (N.L.); 2 UR Magellan, Iaelyon School of Management, University Jean Moulin Lyon 3, 1 Avenue des Frères Lumière, 69008 Lyon, France; jimmy.leblet@univ-lyon3.fr; 3 Laboratoire d’Analyse et d’Architecture des Systèmes (LAAS), Université de Toulouse, CNRS, UPS, 7 Avenue du Colonel Roche, 31031 Toulouse, France; aymen.sendi@capgemini.com (A.S.); menini@laas.fr (P.M.)

**Keywords:** metal oxide gas sensors, nanomaterials, selectivity, temperature modulation, mathematical modeling, data analysis, electronic nose

## Abstract

The need for odor measurement and pollution source identification in various sectors (aeronautic, automobile, healthcare…) has increased in the last decade. Multisensor modules, such as electronic noses, seem to be a promising and inexpensive alternative to traditional sensors that were only sensitive to one gas at a time. However, the selectivity, the non-repetitiveness of their manufacture, and their drift remain major obstacles to the use of electronic noses. In this first work, we show how the mathematical modeling of the sensor response can be used to find new selectivity characteristics, different from those classically used in the literature. We identified new specific characteristics that have no physical meaning that can be used to find criteria for the presence of formaldehyde and nitrogen dioxyde alone or in a mixture. We discuss the limitations of the methodology presented and suggest avenues for improvement, with more precise modeling techniques involving symbolic regression.

## 1. Introduction

The “electronic nose” is a system that simulates the biological nose and its role in odor detection. Odor can be used to identify certain sources of interest or problems. These include air pollution, environmental contamination, disease diagnosis, identification of individuals in criminal investigations, etc. The needs of the various sectors show that multisensor modules, such as electronic noses, appear to be a promising, low-cost alternative for odor measurement, especially when metal oxides are used. However, SnO_2_ gas sensors have two main drawbacks in applications of everyday life: their sensitivity is very low in the sub-20 ppm regime [1] and they are prone to drifts that greatly affect selectivity scores even in public benchmarks [2,3]. The review of He et al. underscores the potential of AI integration for enhanced sensor performance and real-time data analysis [1].

The principle of odor or gas composition identification is inspired by the human nose, through the learning and classification of the identified odor (thanks to the brain), characterized by a response from the sensors (olfactory cells). From a physical measurement system point of view, the aim is to learn to recognize odors through the physical response of microsensors placed in the presence of gas and to determine the presence of target gases [4,5]. This means using a number of different sensors. One way around this problem is to use sensitive metal oxide surfaces at different hotplate temperatures. This reduces the number of sensors required while increasing the number of features available [6,7]. It is thus necessary to characterize the responses of sensitive surfaces at different temperatures during dedicated supervised measurement campaigns in order to be able to identify the gas in a future real situation. The selected microsensors are placed in atmosphere-controlled measurement chambers, where gases are injected, alone or in a mixture, according to a specific measurement protocol. All measurements collected then form a database from which fingerprints are learned using machine learning or deep learning algorithms [8,9,10].

In the literature, selectivity is often expressed after stabilization of the sensor response [6]. For example, the final value of the response to gas injection is a typical selectivity characteristic. However, stability is reached in MOx sensors after ≈2 min, while in real conditions, the variations can be numerous and rapid, making it impossible to wait for the response to stabilize. This is what we are trying to solve by proposing a new method to find selectivity characteristics.

A final major issue concerns sensor drift over time, particularly long-term drift. A great deal of work has been conducted without considering this issue, which distorts the predictive results of the proposed algorithms. In this work, we took account of these issues, as proposed, for example, in the works of Dennler et al. [2] and Chang et al. [3] research teams.

In our study, we chose to focus specifically on MOX sensors based on SnO_2_ nanoparticular sensing materials, deposited on a micro-machining micro-hotplate with platinum interdigitated electrodes and a platinum heating resistor. Volatile compounds or gases react with the metal oxide surface by chemisorption (activated by temperature) and then cause a resistivity change [4,5,6,8,9,10].

The aim of this initial work is to find an automatic calibration method to selectively identify some chemical compounds without having to take into account drift and manufacturing faults on sensitive surfaces. Our work is based on laboratory-produced sensitive layers that exhibit imperfections, whose performance measurements are to be found in [7].

First, we will describe how the sensitive surfaces studied were created. Then, we will present a selectivity research methodology that normalizes sensor drift and calibration in terms of target resistivity values. We will then present the obtained results and the relevance of our methodology. Finally, we will discuss possible improvements to our methodology.

## 2. Materials and Methods

### 2.1. SNO_2_ Sensor Description

The structure is characterized by its circular membrane and comprises two levels of Ti/Pt metallization: one in a circular coil for the heater and the other in circular, interdigitated measurement electrodes, superimposed and separated by a passivation layer [7].

The sensitive layer studied in this work is based on SNO_2_ nanoparticles. The protocol used to produce it is the one described in Aymen Sendi’s thesis [7]. SnO_2_ nanoparticles were obtained by reacting controlled quantities of water (8 molar equivalents) on the bis(dimethylamido) tin (II) precursor [(Sn((NMe)_2_)_2_]_2_ in the presence of dodecylamine (C_12_H_27_N) (DDA) (10 molar equivalents). The solution was left for 4 days in synthetic air at room temperature in a pillbox to obtain Sn_2_O_2_(OH)_2_ nanoparticles as a white powder. This powder was washed with THF to remove excess ligands before being calcined in a furnace under ambient air at 350 °C or 500 °C to form SnO_2_. The calcined powder is used directly to produce a screen-printing paste for SnO_2_-based sensors.

**Figure 1:**
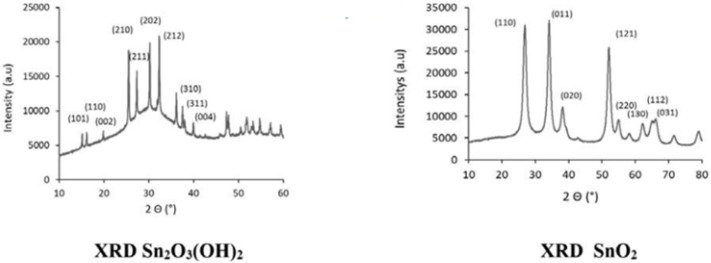
Characterization of Sn_2_O_3_(OH)_2_ and SnO_2_ powder by X-ray diffraction before and after annealing and comparison of grain sizes from XRD.

The powder obtained was characterized by X-ray diffraction before and after annealing at 500 °C. Before calcination, the phase present was tin oxohydroxide Sn_3_O_2_(OH)_2_ presented on the left of Figure 1. After calcination in air, only the cassiterite phase of SnO_2_ is present on the right of Figure 1.

**Figure 2:**
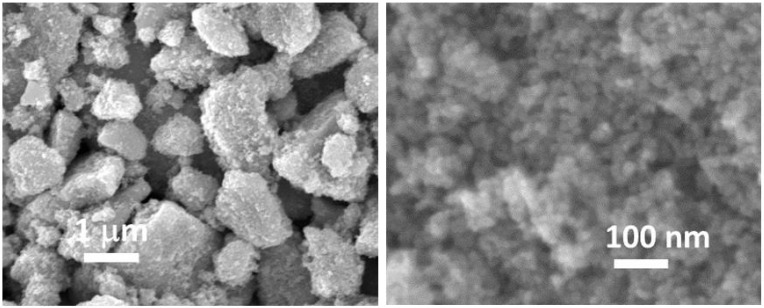
SEM images of SnO_2_ nanoparticles produced, showing agglomerate and nanoparticle arrangements, respectively.

The SnO_2_ nanoparticles were observed by SEM. The powder is in the form of agglomerates of the order of a few microns in size, as shown on the left of Figure 2. Each agglomerate is itself made up of nanoparticles less than 10 nm in diameter, as shown on the right of Figure 2.

Let us now present how we used these sensors in our experiment.

### 2.2. Experimental Set-Up

The micro-hotplates were designed and manufactured in LAAS-CNRS. Experimental characterization of the sensor and comparative tests with reference instruments can be found in [6] (pp. 67–112). It is optimized to operate at a high temperature with low power consumption, 500 °C at 50 mW, and a low temperature, 100 °C at 10 mW with low thermal inertia (20 ms) and high thermomechanical stability [11].

In order to analyze the response to this sensor and test our new selectivity detection methodology, we applied a specific gas injection protocol in a test chamber containing the sensor. For each injection of gas, gas mixture, or air, we forced the heater to first maintain a temperature of 500 °C and then 100 °C for 150 s each. For each injection, this step is repeated 6 times.

**Figure 3:**
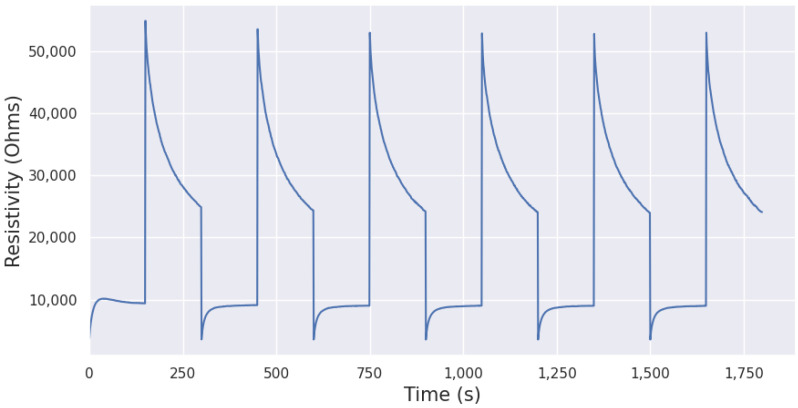
Nanoparticular SnO_2_ response in Ohms under formaldehyde (2 ppm) successively at 500 °C and 100 °C.

As an example, the response to an injection of formaldehyde (2 ppm) according to the protocol described is shown in Figure 3.

The gas injections were performed in the order described in Table 1. Concentrations are expressed in ppm and are in range with industrial sensors as TGS 2620 (50 ppm for CO). The different atmospheres are made with a constant total flow rate of 200 mL/min controlled by digital flowmeters.

Now that we can measure the response of various gases, let us present how we mathematically model them.

### 2.3. Mathematical Modeling of Sensor Response

The sensitive surface’s response to a gas injection has a specific transient response depending on its type (Figure 3), the type of gas injected, and the temperature variation of the micro-hotplate. We can see the response has a recurring pattern for each temperature step. Our goal is to mathematically model these two patterns for each gas, allowing us to infer enabling characteristics for selectivity clustering algorithms, described in Section 3.3.

For this very first work, in order to take into account the stabilization of the response in dynamic mode, we consider the last dynamic sequence (extracted thanks to the experiment timestamps) at 500 °C and 100 °C for each gas injection.

To avoid sensor drift issues and the initial non-stabilized response for each new gas sequence, we normalize all the responses under gas with the values of the previous sequence in the reference atmosphere (humid air) [2,3]. In future work, we will be adding an algorithm for the automatic detection of modeling changes so that we can normalize using the previous response without having to go through the under-air sequence.

In terms of modeling, previous work in the LAAS-CNRS laboratory used piecewise affine modeling to describe the sensor response to a gas injection [6]. However, this approach is a large approximation, which does not take into account all the information available in the data, which is why we propose, in this paper, to mathematically model all sensor responses for each injection of gas and gas mixtures with more elaborated models given in the next section.

This initial modeling work was carried out by manually testing a series of common mathematical functions, such as logarithmic, exponential, polynomial, or rational functions, expanding over the polynomial fitting of [12]. The model selection presented in the following section was solely made on visual and subjective criteria. Nonetheless, these first models allowed us to develop a new methodology for finding selectivity criteria, which is the point of this initial work. We will discuss in Section 4.2 a systematic way to derive models with precision for our future work.

### 2.4. Machine Learning Strategy

The last brick of our methodology is a machine learning algorithm that will help us identify which parameters are selective for our gas selectivity problem. Here are the most commonly used methods. They are listed in order of complexity and are usually tested in this order: if one fails to give relevant results, the next one is tried. See [13] for more details.

- Principal Component Analysis (PCA) consists of a dimensionality reduction in order to summarize the information content of a wide range of features. This gives a new graphical representation that allows for better qualitative classification. However, this classification must be performed by a human so it must be complemented by machine learning tools for better objectivity.
- Linear Discriminant Analysis (LDA) tackles this problem by learning from a dataset to label the data points. Like PCA, it also reduces feature dimensionality but maximizes the separation of groups of similar points.
- Support Vector Machines (SVMs) are a set of supervised learning techniques whose aim is to find the hyperplane that best divides a dataset in two. They are particularly interesting because their results are easily embedded in electronic noses.
- Artificial Neural Networks (ANNs) are a great tool to process and analyze non-linear data. When trained with labeled data, they are able to recognize patterns, which is a critical task in electronic noses for odor recognition for instance.

In this first work, we chose to stick with the PCA and SVM methods, as the core of our contribution relies on the innovative modeling of the sensor’s response.

Let us now present the results of this new methodology.

## 3. Results

### 3.1. New Selectivity Criteria Derived by Mathematical Modeling of $SnO_{2}$ Sensor Response

We recall that 60 injections were made in the order given in Table 1. Since the sensor’s response was similar for all gases and mixtures, we searched for a single common model for each temperature only. The mathematical model adopted for all the responses to gas alone or in mixture for temperatures of 500 °C and 100 °C, respectively, are

(1) $$\begin{array}{ccc} f_{500}\left(x\right) & = & \alpha +\frac{1}{u*x+w}, \end{array}$$

(2) $$\begin{array}{ccc} f_{100}\left(x\right) & = & a*\ln(b*x+c)+d. \end{array}$$

**Figure 4:**
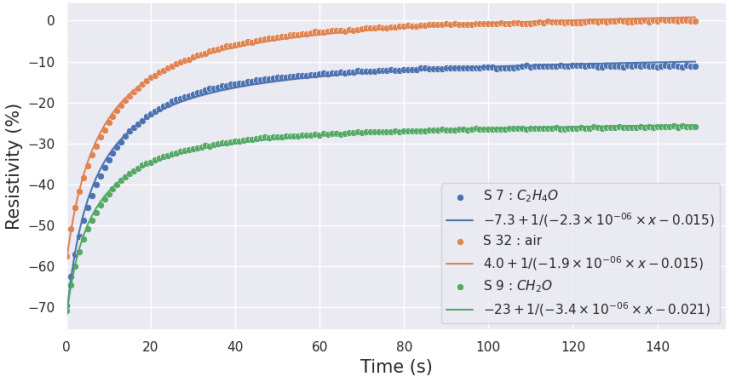
Acetaldehyde, air, and formaldehyde response modeling at 500 °C (continuous line) against the data (dots).

**Figure 5:**
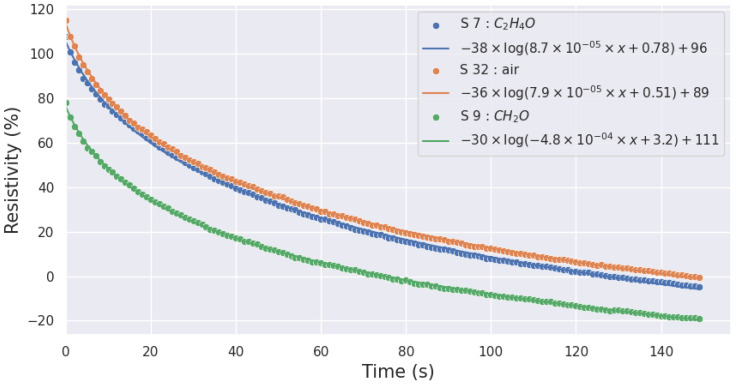
Acetaldehyde, air, and formaldehyde response modeling at 100 °C (continuous line) against the data (dots).

For each injection, we computed the coefficients by using non-linear least squares to fit the function to the data. As an example, Figure 4 and Figure 5 show the quality of the modeling of the resistivity of the SnO_2_ layer in response to the presence of acetaldehyde, air, and formaldehyde for a heating temperature of 500 °C and 100 °C, respectively.

From our models, the characteristics we derived and were retained as selectivity search criteria are given in Table 2:

**Table 2:** The 11 features used for our selectivity analysis.

Temperature	Features					
100 °C	*d*	*b*	*c*	*a*	${\mathrm{vfn}}_{100}$	$f_{100}^{\prime }\left(0\right)$
500 °C	α	*u*	*w*		${\mathrm{vfn}}_{500}$	$f_{500}^{\prime }\left(0\right)$

where

- ${\mathrm{vfn}}_{100}$ and ${\mathrm{vfn}}_{500}$ are defined as the average of the three last points of the sequence (the asymptotic value). The sensor’s response time being around 2–3 min, it is hardly available for detecting a gas composition change, which is represented by a peak lasting a few seconds. vfn is used here to test the relevance of this choice proposed in some articles in the literature in terms of the selectivity criteria [6,14].
- $f_{100}^{\prime }\left(0\right)$ and $f_{500}^{\prime }\left(0\right)$ are the slope at the origin of the sequence, also used in other articles [14].

**Figure 6:**
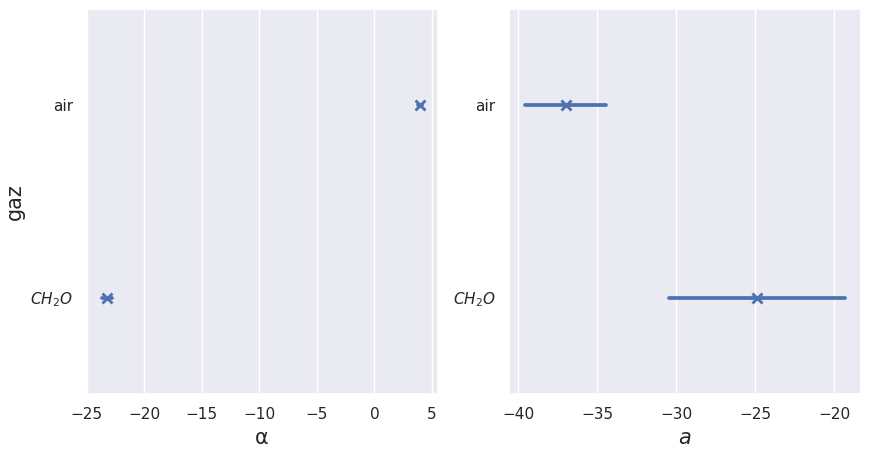
Characteristic *a* and α derived from curve fitting of all available sequences of all cycles.

Before applying machine learning tools for clustering, we checked that measurement uncertainty on the coefficients did not hinder their use as selectivity criteria. As an example, we present here the measurement error coefficients $a_{norm}$ and $\alpha_{norm}$ for formaldehyde and reference air. Figure 6 shows the measurement error bars for characteristic *a*, respectively, α for air and formaldehyde. We chose to represent standard deviation error bars. It corresponds to the average distance between each data point and the mean. These results show that uncertainties do not call into question the use of these characteristics for selectivity since there is no overlap of values.

Now that we have our selectivity criteria and our coefficient values from our mathematical models, let us use machine learning tools in order to analyze them.

### 3.2. Classical Selectivity Testing Method: Principal Component Analysis

As suggested by the literature [8,10,12], we decided to use the PCA method as a first approach, given the number of features involved.

**Figure 7:**
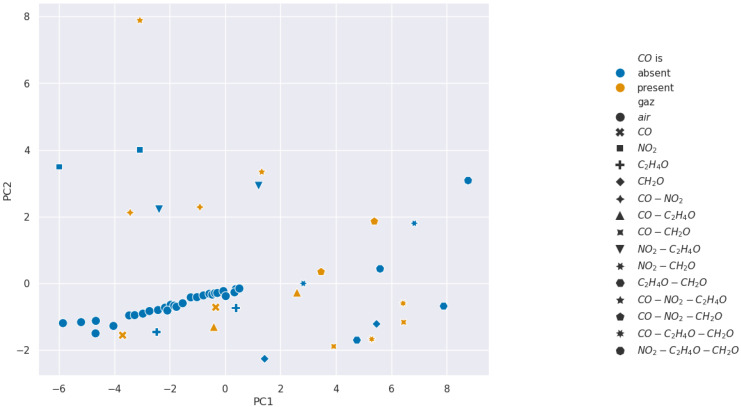
Projection over the first two Principal Components retrieved from a PCA for carbon monoxide selectivity analysis.

**Figure 8:**
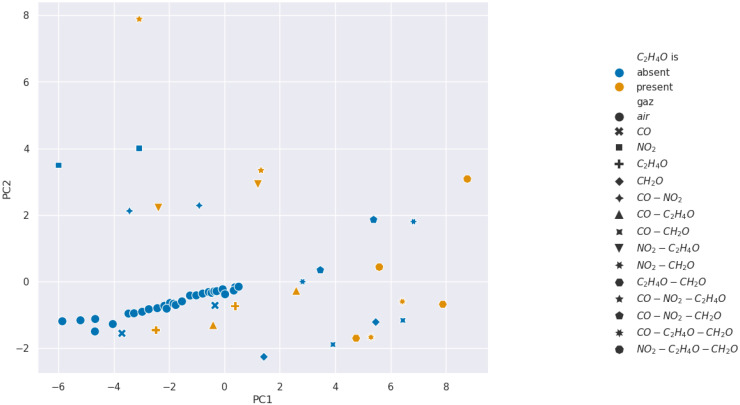
Projection over the first two Principal Components retrieved from a PCA for acetaldehyde selectivity analysis.

**Figure 9:**
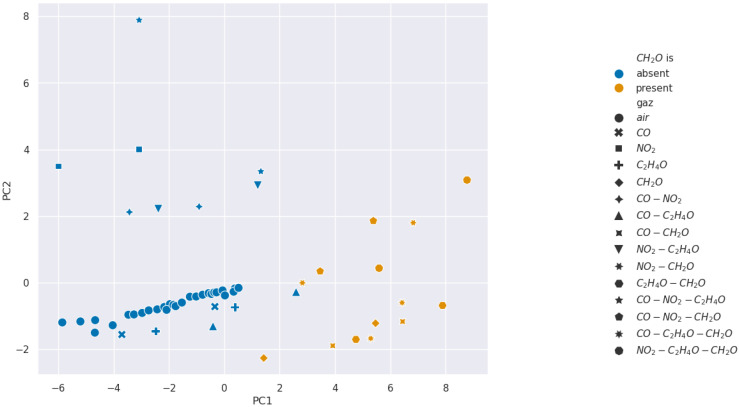
Projection over the first two Principal Components retrieved from a PCA for formaldehyde selectivity analysis.

**Figure 10:**
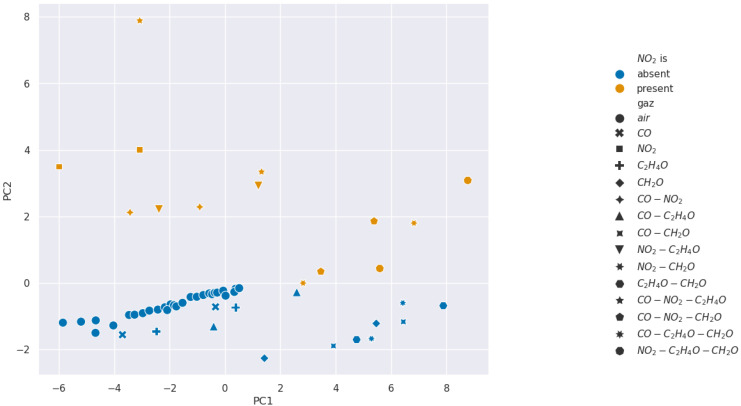
Projection over the first two Principal Components retrieved from a PCA for nitrogen dioxide selectivity analysis.

We present the PCA analysis in Figure 7, Figure 8, Figure 9 and Figure 10. In these Figures, each point represents a given injection from Table 1. Moreover, the color describes the concentration of the gas we want to detect: in blue it is absent, and in orange it is present.

The results show that selectivity for acetaldehyde and carbon monoxide is not identifiable from the PCAs performed on our 11 features from Table 2. However, clusters appear for formaldehyde and nitrogen dioxide, which suggests the existence of relevant features for the selectivity of these gases. Consequently, we wanted to analyze features in pairs using SVM methods to find more separated clusters.

### 3.3. Specific Support Vector Machines

We recall that our main goal is to increase the sensors’ gas selectivity even if the sensitive surface may be flawed. Already known selectivity features are the derivatives at the beginning and the asymptotic values of the gas response. Using our mathematical models and the associated parameters, we wish to find the best features for gas selectivity. In order to do that, we define the problem as a machine learning task. We define the selectivity of a target gas in a mixture as a binary classifier, where sequences have True labels if the gas is in a mixture and False otherwise. For example, sequence 55 (c.f. Table 1) would have True labels for classifying CO, C_2_H_4_O, and CH_2_O, and False for NO_2_.

For a binary classifier, the metric of choice to determine its quality is the F1-score:

(3) $$\text{F1-score}=\frac{\mathrm{True}\;\mathrm{Positives}}{\mathrm{True}\;\mathrm{Positive}+\frac{1}{2}\left(\mathrm{False}\;\mathrm{Negative}+\mathrm{False}\;\mathrm{Positive}\right)}$$

**Figure 11:**
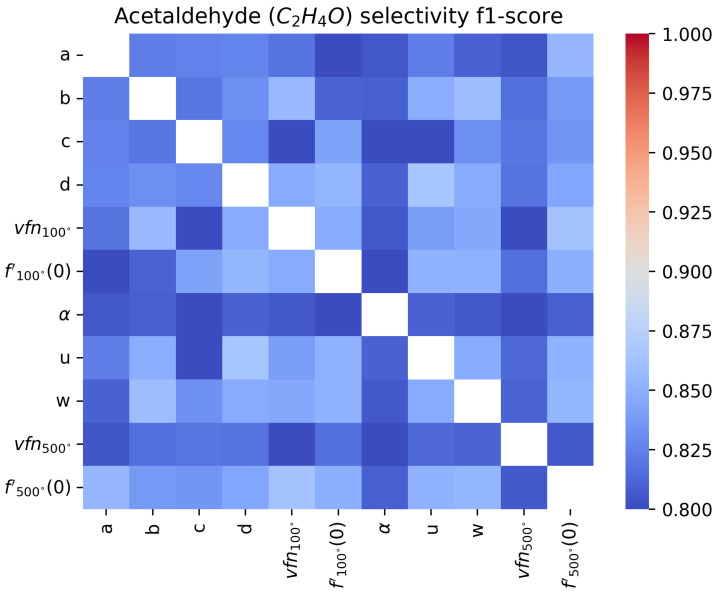
Comparison of F1-score of a binary classifier SVM over the acetaldehyde sequences over the extracted features for each pair of features.

**Figure 12:**
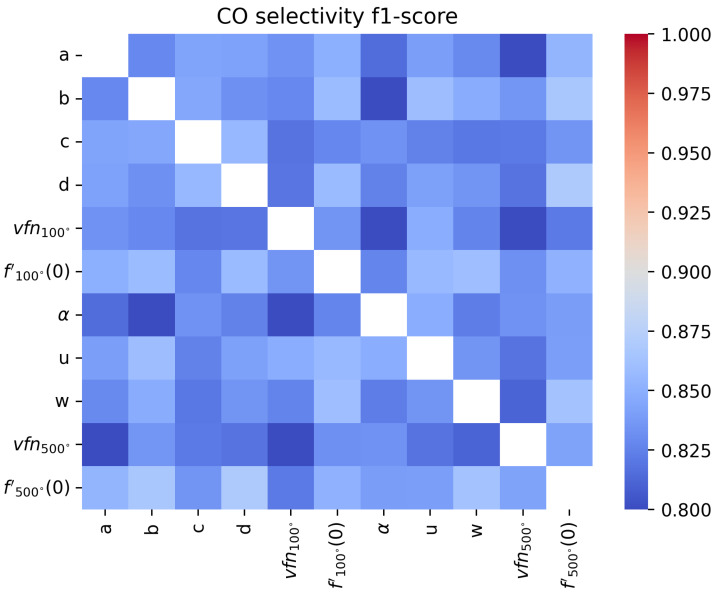
Comparison of F1-score of a binary classifier SVM over the carbon monoxide sequences over the extracted features for each pair of features.

**Figure 13:**
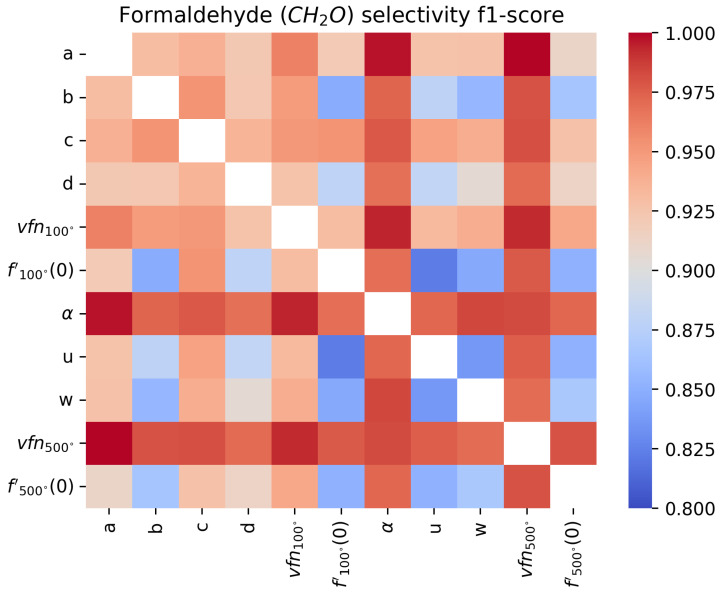
Comparison of F1-score of a binary classifier SVM over the formaldehyde sequences over the extracted features for each pair of features.

**Figure 14:**
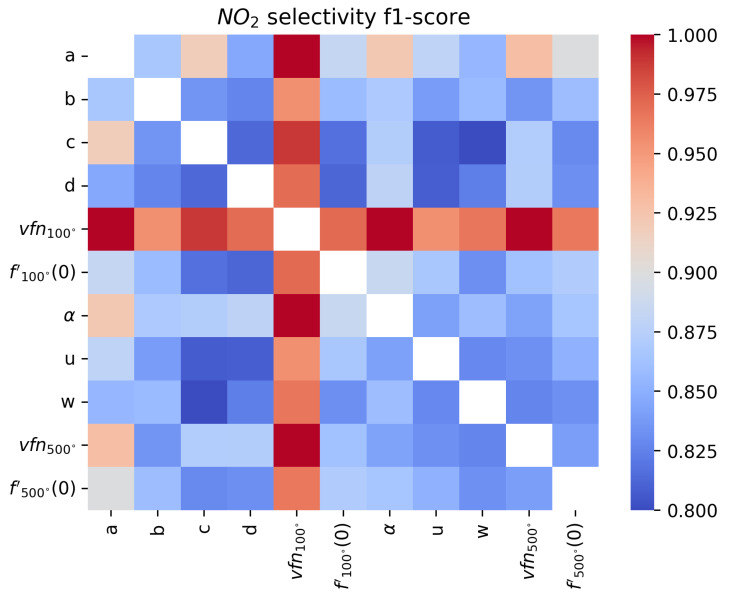
Comparison of F1-score of a binary classifier SVM over the formaldehyde sequences over the extracted features for each pair of features.

An F1-score close to 1 means that the sensor’s sensitivity with respect to the target gas is perfect, while an F1-score close to 0.8 means the features do not allow for sensitivity or that the surfaces are not selective since the score would correspond to a fully random classifier. In Figure 11, Figure 12, Figure 13 and Figure 14, we show the results for a Support Vector Machine binary classifier with a linear kernel for each pair of features and each gas for all the sequences in Table 1. Using this matrix representation, it is easy to find which pair of features from our mathematical modeling at 100 ºC and 500 ºC are the best if several features are colored in red.

**Figure 15:**
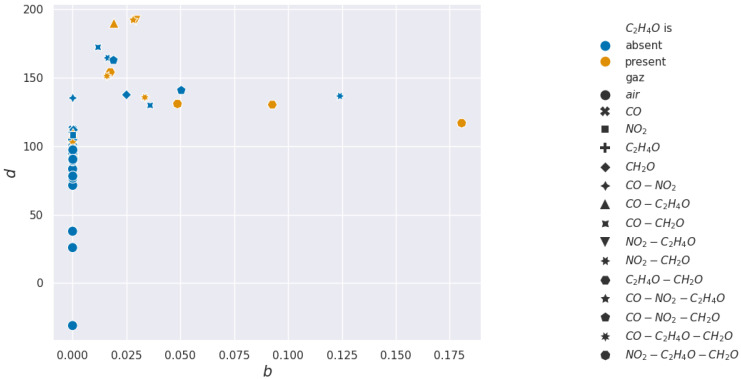
Acetaldehyde projections over *d* and *b* used for the Support Vector Machine learning.

**Figure 16:**
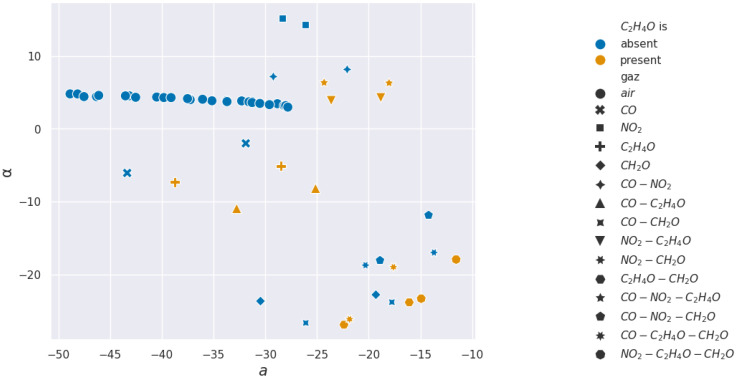
Acetaldehyde projections over α and *a* used for the Support Vector Machine learning.

For acetaldehyde and carbon monoxide, this method of clusterization based on retained characteristics does not allow separation. The layer studied, according to the methods presented, is not selective to these two gases. In Figure 15 and Figure 16, two pairs of features are shown to illustrate how hard it visually is to discriminate between sequences with or without acetaldehyde.

For NO_2_, we find that the most meaningful feature is the asymptotic value ${\mathrm{vfn}}_{100}$, as commonly found in the literature [6]. But for real-time applications, where the sensor temperature switches constantly, it can be hard to measure it. This matrix representation shows that α and *a* can be good features for NO_2_ sensitivity instead. To improve the sensitivity, it will be important to find other features or improve the modeling, as we will discuss in Section 4.2.

**Figure 17:**
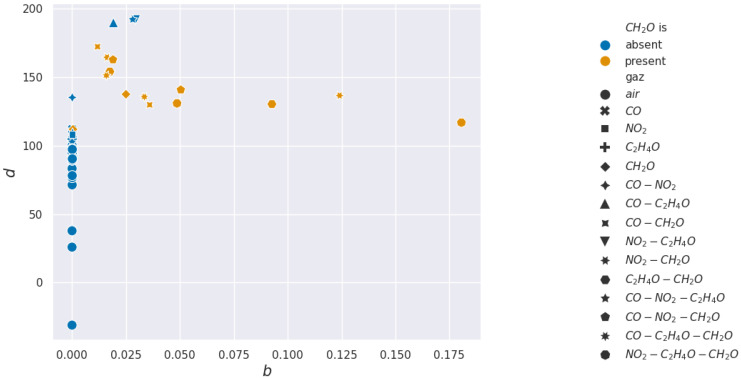
Formaldehyde projections over *d* and *b* used for the Support Vector Machine learning.

**Figure 18:**
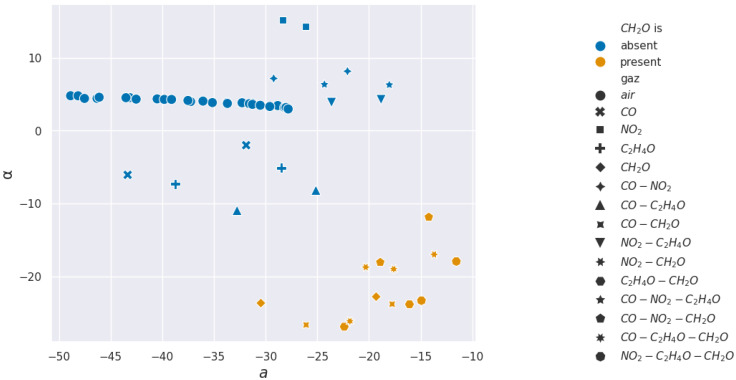
Formaldehyde projections over α and *a* used for the Support Vector Machine learning.

In the formaldehyde case, almost all features can detect its presence, with α being the best feature to use for real-time detection. In Figure 17 and Figure 18, two pairs of features are shown to illustrate how easy or hard it visually is to discriminate between sequences with or without formaldehyde.

We conclude this article with a discussion of the results and how to improve our new methodology on several points for future works.

## 4. Discussion

In this paper, we aim to propose a new approach for extracting selectivity characteristics even when sensitive surfaces have defects in order to minimize the impact on the production of minor surface defects that sometimes drastically alter the sensor response. The results we obtained in this initial work are very encouraging, but there is still room for improvement.

### 4.1. Clustering Approach

We showed that the methodology presented allows us, using SVM, to find the best selectivity criteria derived from the derived characteristics of the proposed formaldehyde detection model.

Acetaldehyde and CO gases have no meaningful results with respect to our experiment and mathematical modeling. Here are some possibilities for this insensitivity:

- The Support Vector Machine is not good enough for some clustering, as for example in Figure 15 where another kind of separation like K-Means could be better.
- The sensors are not selective with respect to those gases. The results hence could show that there is a need for other surfaces than the used micro-hotplate SnO_2_.

### 4.2. A New Approach to Derive Precise Mathematical Models

The results shown in this paper are derived from the coefficients of Equations (1) and (2), which were found by trying many different functional forms by hand and selecting the most promising candidates, and then optimizing the value of its constants to fit the data points. We believe that a better physical model would improve the accuracy and sensitivity of the method. Even though many theoretical models exist [15] for different kinds of sensors and gases, finding good candidates can get very tedious. For example, Mitchell et al. [9] sorted multiple candidates by hand based on physical equations.

Symbolic regression (SR) [16,17] is a type of machine learning regression analysis that searches for mathematical expressions to model relationships within a dataset. Unlike traditional regression methods that fit data to a predefined equation, SR explores a broad space of potential mathematical forms, automatically identifying both the structure and parameters of the equation. This approach enables it to discover compact, interpretable models that capture underlying patterns, making it valuable for scientific research where the goal is to unveil fundamental, interpretable relationships rather than just achieving predictive accuracy. At Capgemini Engineering, we developed Newton-SR, a symbolic regression tool based on genetic programming to find the best candidates, and won the SR Bench 2023 competition with open-source code [18].

We will aim at generalizing the analysis performed in Section 3 using our SR algorithm. This would allow for faster identification of the various gases and open new possibilities in sensor calibration in the long run. This will be the object of our next article. In the meantime, here is an example of the sequence presented in Section 2.3. The equation found by hand () has the following parameters when fitted to the data:

(4) x↦−30.46∗ln(0.000483∗x+3.25)+111.96.

In comparison, the functions generated by our algorithm are given in Table 3. We define here the several metrics used to compare several functions:
  - Mean Square Error (MSE): the average distance between the data points and the function for a cloud of points $E:={\left\{\left(x_{i},y_{i}\right)\right\}}_{i=1}^{N}\subset R^{d}\times R$ and a function *f* is defined as
    $$MSE\left(f\right)\left(E\right):=\frac{1}{N}\sqrt{\sum_{i=1}^{N}{\left(y_{i}-f\left(x_{i}\right)\right)}^{2}}.$$
  - Complexity: The number of operators in the tree representation of a function. For instance, x↦x+1 has a complexity of three.
  - Efficiency: the MSE log gain relative to the previous less complex function.

As a result, (4) has a complexity of 10 and an MSE of 0.42. Using symbolic regression, the function of complexity nine in Table 3 is less complex and has a better MSE of 0.235, thus improving accuracy without compromising the complexity of the representation:

(5) $$x\mapsto \frac{500,882.000}{3196.380+x^{0.719}}-78.678$$

## 5. Conclusions

The aim of our work is to propose a methodology to overcome defects in the selectivity of sensitive layers and the well-known time drift of this type of sensitive surface. In conclusion of this first work, we can state that the mathematical modeling of sensor responses to single or mixed gases allows for a good selectivity with only a couple of features compared to higher numbers used in other works. We showed that specific characteristics derived from modeling that have no physical meaning a priori, such as *a* and α defined in (2) and (1), can be used to find criteria for the presence of formaldehyde alone or in a mixture. We discussed the limitations of the methodology presented and suggested avenues for improvement, with more precise modeling techniques involving symbolic regression and new sensors from the market. This work is ongoing and will be the subject of a forthcoming publication.

## Figures and Tables

**Table 1:** Ordered list of gas injections in the experiment with their concentration in ppm.

S	Gas Mixture	CO	NO_2_	C_2_H_4_O	CH_2_O
2	Air	0	0	0	0
3	CO	50	0	0	0
4	Air	0	0	0	0
5	NO_2_	0	0.2	0	0
6	Air	0	0	0	0
7	C_2_H_4_O	0	0	0.2	0
8	Air	0	0	0	0
9	CH_2_O	0	0	0	2
10	Air	0	0	0	0
11	CO, NO_2_	50	0.2	0	0
12	Air	0	0	0	0
13	CO, C_2_H_4_O	50	0	0.2	0
14	Air	0	0	0	0
15	CO, CH_2_O	50	0	0	2
16	Air	0	0	0	0
17	NO_2_, C_2_H_4_O	0	0.2	0.2	0
18	Air	0	0	0	0
19	NO_2_, CH_2_O	0	0.2	0	2
20	Air	0	0	0	0
21	C_2_H_4_O, CH_2_O	0	0	0.2	2
22	Air	0	0	0	0
23	CO, NO_2_, C_2_H_4_O	49	0.2	0.15	0
24	Air	0	0	0	0
25	CO, NO_2_, CH_2_O	50	0.2	0	2
26	Air	0	0	0	0
27	CO, C_2_H_4_O, CH_2_O	50	0	0.2	2
28	Air	0	0	0	0
29	NO_2_, C_2_H_4_O, CH_2_O	0	0.2	0.2	2
30	Air	0	0	0	0
31	CO	50	0	0	0
32	Air	0	0	0	0
33	NO_2_	0	0.2	0	0
34	Air	0	0	0	0
35	C_2_H_4_O	0	0	0.2	0
36	Air	0	0	0	0
37	CH_2_O	0	0	0	2
38	Air	0	0	0	0
39	CO, NO_2_	50	0.2	0	0
40	Air	0	0	0	0
41	CO, C_2_H_4_O	50	0	0.2	0
42	Air	0	0	0	0
43	CO, CH_2_O	50	0	0	2
44	Air	0	0	0	0
45	NO_2_, C_2_H_4_O	0	0.2	0.2	0
46	Air	0	0	0	0
47	NO_2_, CH_2_O	0	0.2	0	2
48	Air	0	0	0	0
49	C_2_H_4_O, CH_2_O	0	0	0.2	2
50	Air	0	0	0	0
51	CO, NO_2_, C_2_H_4_O	40	0.2	0.2	0
52	Air	0	0	0	0
53	CO, NO_2_, CH_2_O	50	0.2	0	2
54	Air	0	0	0	0
55	CO, C_2_H_4_O, CH_2_O	50	0	0.2	2
56	Air	0	0	0	0
57	NO_2_, C_2_H_4_O, CH_2_O	0	0.2	0.2	2

**Table 3:** Best-fitting candidates found for the sequence presented in Section 2.3 using symbolic regression, sorted by complexity. *x* represents the time dynamics. The best equation is defined as the one that minimizes efficiency.

MSE	Complexity	Efficiency	Equation
0.208	19	−0.008	((x/−6840.830) + (−57,955.400/(−3064.320 − x)) + (4,586,540.000/((106,033.000 * exp((x/48,836.400))) − 28,663.100)))
0.209	18	−0.004	((173,644.000/(2218.140 + (((x ** 2.415)/704,367.000)/109.105) + (x ** 0.772))) − (x/6563.040))
0.210	17	−0.004	((4,857,520.000/(x + 49,061.900 + (−19,350,100.000/(x + 3016.620)))) − (35.652 * exp((x/724,710.000))))
0.211	16	−0.061	(((4,643,410.000/(x + 47,861.400 + (−18,701,000.000/ (x + 2972.830)))) − (x * 0.000)) − 33.448)
0.224	15	-0.017	((977,649.000/(6862.460 + (x ** 0.791) + (−1.000 * ((−604,512.000/(−2231.820 − x)))))) − 70.013)
0.232	13	−0.004	(((x * 0.000) + (506,206.000/(2980.680 + (x ** 0.702)))) − 91.603)
0.235	9	−0.758	((500,882.000/(3196.380 + (x ** 0.719))) - 78.678)
0.502	8	−0.752	(−369.368 − (−4201.520/log((x + 13,003.900))))
1.066	7	−1.999	((5,449,210.000/(x + 46,945.000)) − 45.749)
7.864	6	−0.000	(x + 43.101 + (x/−1.000))
7.864	5	−1.032	(0.000 * (88,065.400 − x))
22.072	4	−0.008	(89.100/log(x))
22.604	1	0.000	6.639

## Data Availability

Data are contained within the article.
